# Visualising Medical Research: Exploring the Influence of Infographics on Professional Dissemination

**DOI:** 10.1155/2024/5422121

**Published:** 2024-06-18

**Authors:** Sujin Butdisuwan, Lovely M. Annamma, A. Subaveerapandiyan, Biji Thomas George, Sanjay Kataria

**Affiliations:** ^1^Faculty of Psychology, Metharath University, Pathum Thani 12160, Thailand; ^2^Department of Education, Educational Administration, INTI International University and Colleges, Nilai, Malaysia; ^3^Department of Clinical Sciences, College of Dentistry, Ajman University, Ajman, UAE; ^4^Department of Library, Sai University, One Hub Road Paiyanur, Chennai 603104, Tamil Nadu, India; ^5^Department of Library, Bennett University, Greater Noida, India; ^6^RAK College of Medical Sciences (RAKCOMS), RAK Medical & Health Sciences University (RAKMHSU), P.O. Box 11172, Ras Al Khaimah, UAE; ^7^Department of Library, Bennett University, Greater Noida, India

## Abstract

**Objective:**

This study explores the impact of infographics on the professional dissemination of medical research. Recognising the burgeoning volume of data in the medical domain, this research aims to bridge the gap by investigating the efficacy of infographics in rendering complex medical concepts understandable to diverse audiences, including policymakers, patients, and the public.

**Design:**

The study uses a cross-sectional survey to assess medical professionals' familiarity with infographic design and data visualisation principles. *Setting*. The research targets medical professionals with published articles across various subfields, including Clinical Medicine, Epidemiology, Pharmacology, Healthcare Management, Medical Imaging, and Public Health.

**Method:**

Data collection involves an online survey distributed to potential participants through professional networks and research institutions. The survey encompasses Likert-scale questions and demographic variables. Ethical considerations include obtaining approval from the institutional review board, ensuring participant consent, and maintaining data anonymity and confidentiality.

**Results:**

Demographic analysis reveals a diverse participant profile, with 58.7% male and 41.3% female respondents, spanning various age groups, professional experiences, and geographic locations. Assessing familiarity with infographic design and data visualisation principles demonstrates respondents' proficiency in certain areas while highlighting potential areas for improvement.

**Conclusion:**

The study underscores the multifaceted benefits of infographics in research dissemination, as medical professionals perceive. Infographics can effectively convey various kinds of medical research information across diverse platforms and channels.

## 1. Introduction

Effective communication of research findings is a dynamic challenge in today's ever-evolving landscape. Traditional methods of conveying research results often rely on extensive textual formats, which might not engage modern audiences. To address this issue, innovative visual communication tools, such as infographics, have emerged as potent vehicles for presenting complex information concisely and captivatingly [1]. The term infographics refers to the visual representation of information or data. They distil intricate data and concepts into visually appealing and easily understandable formats, offering a dynamic alternative to text-heavy approaches [2–4]. The various types of infographics used in the medical field are statistical infographics that represent visual communication through graphs, charts, or percentages. This helps to quickly understand the correlations, patterns, and trends which can be missed while reading from within a text. The following common types of infographics used are process infographics, which depict the breakdown of information in linear, easy-to-understandable patterns. Comparison infographics aid in absorbing the differences between trends. Hierarchical, timeline, interactive, flowchart, map, and visual abstracts are a few other types of infographics [5, 6].

Infographics trace back to ancient times when cave-dwelling communities utilised visual representations for communicating ideas, especially without written language [7]. Over the past millennia, infographics have reemerged as a powerful means of conveying information swiftly and effectively [8]. A single image possesses the capacity to convey a multitude of meanings contingent on individual perceptions [9]. When integrated with the text, the context of the information provided by the infographics is routinely used in public education, as in traffic signals and scientific fields, for clarity [10, 11].

Information visualisation has assisted information seekers [12, 13] and is used to effectively cater to the community's diverse needs. To ensure that the most critical findings are conveyed to the audience most efficiently, infographics are utilised during conferences and poster presentations [14], on social media platforms, in education, and even for amusement [15–17]. Libraries and librarians utilise infographics extensively for crafting user guides, elucidating library services, software utilisation, and metric study tools [18, 19].

In research, infographics are essential for effectively communicating complex information [20]. Their ability to convey complex information concisely and engagingly has revolutionised how medical insights are disseminated [21]. This paper focuses on exploring the transformative potential of infographics in the context of disseminating medical research. Through a meticulous cross-sectional analysis involving medical experts, this study aims to uncover the multifaceted impacts of infographics on conveying essential medical insights.

### 1.1. Literature Review

Infographic use in the medical field was reported by Martin et al. [22] and observed to have the advantages of overcoming language barriers and enhancing medical information accessibility. It examines five infographic types: Isotype Arrays for unbiased representation, Polar Area Diagrams for temporal trends, Word Clouds for text summarisation, Hub and Spoke Diagrams for complex relationships, and Charticles for concise communication. Infographics offer a promising approach, but the study emphasises the need for precise design and context, particularly for diverse audiences. Infographics effectively combine storytelling, creative visual design, and efficient technological utilisation [23]. Infographics represent literature with text and visual information. In multimedia worlds, visual description is widely recommended [24, 25]. The infographics can be in the form of diagrams, graphs, images, and text. This is widely used in pedagogy methods [26].

During the past few years, visual data applications have been used to convey critical information in the scientific literature [27]. Visual data can be erroneous if the data collection technique or data assimilation is not meticulously followed. Cleveland [28] discovered that around 30% of the graphs featured in the journal Science contained at least one form of error. Infographics can only be effective if data are properly organised [29].

Damschroder et al. [30] observed in their studies that much of the medical research documented as evidence-based is not translated into patient care and safety outcomes. Hence, they composed five major domains: intervention characteristics (e.g., evidence strength and quality), outer setting (e.g., patient needs and resources), inner setting (e.g., culture and leadership engagement), characteristics of the individuals involved, and the process of implementation (e.g., plan, evaluate, and reflect). They observed that following this composed structure could enable evidence-based publication to translate into practice in the medical field. The same structure can be used for other organisations to implement publications in practice.

Nieuwland et al. [31] observed that the most commonly used knowledge dissemination methods were academic journals, conferences, reports to funders, and social media. The above dissemination required resources, knowledge, skills, and finance. Regarding public news and social media, workshops and meetings were better suited for the public [31, 32]. Balkac and Ergun [33] observed that using infographics helps the public and health sectors understand and grasp the information quickly without much complication, provided the graphics are aesthetic and engaging. Infographics can be used in healthcare, technology, business, and entertainment. For an excellent organisation of an infographic, Midway's [27] ten principles are practical. The principles include prioritising information over design, using suitable software, choosing appropriate geometries, using colour strategically, incorporating uncertainty, and employing small multiples for comparisons. For clarity, a combination of simple visuals with detailed captions is significant. An external opinion for feedback on the created infographics is a valuable tool for assessing content efficiency. These principles guide the creation of impactful visuals that convey insights accurately and memorably.

Martin et al. [22] compared reader preference and delayed information retention between infographic article summaries and traditional text-only research abstracts. Participants preferred infographics with lower cognitive load for reader preference. Many evaluations based on eye tracking have been used to evaluate whether infographics interest readers more. The parameters include total fixation duration (TFD), which measures the reader's interest within an area where the cognitive effort by the reader is measured [34]. Another parameter measure was gaze duration (GD), which represents the reader's ability to grasp concepts. A more extended gaze indicates a longer time to understand the concept [35]. If images or diagrams are used coherently, viewers find it easier to grasp the matter than reading through the exact content text [36].

Among the types of studies related to graphical representation, the interrelationship between visuospatial working memory (VSWM), verbal working memory (VWM), and executive functioning (EF) has been studied by many authors. Of the three, the VSWM was observed to be more memory-retentive and had more comprehension in readers [37, 38]. The basics behind the reason graphics are more retentive and accessible to grasp are based on the theory of decreasing cognitive load. The cognitive load can be reduced by decreasing the intrinsic, extraneous, and germane load. Intrinsic is related to the complexity of the topic, extraneous refers to external factors that distract the learning process, and germane load indicates the mental load expended to understand the topic [39]. The reason that graphics are processed better is that the brain processes information through a verbal channel and a nonverbal channel. Hence, the brain stores information dually, so retention regarding the topic will be better for the reader. Graphic posts need to be to the point to avoid extraneous load [40].

The advantages of infographics include pandemic situation education [41] and advertisements on social media for more coverage by retweeting or reposting [42–45]. Many medical conferences are presented to attendees in the infographic method [46–48]. Many design programs, such as Piktochart and Visual.ly, are available to make graphic abstracts [49]. It is informative for patient education and counselling [50]. Infographics can also be used as an experimental tool in researching students' cognitive psychology [51].

Graphical abstracts added to the abstract were found to be more liked by readers, as many authors have reported that graphical abstracts had more usage than abstracts without graphics [52]. Graphical abstracts increased readers' attention to the manuscript [42, 52, 53]. Research of randomised controlled trials using graphic abstracts increased the chance of readability of that specific article three times more . In educating students, Providenza et al. reported that student satisfaction and understanding were enhanced [3]. When the theory classes are supported by visual media, the comprehension of the subject is more than the explanation of the theory alone . Podcasts with audio mediums supplemented by video are also used for medical education and research dissemination [48]. Collaborative platforms such as Google Drive or Dropbox can be used to share infographics within research teams or with collaborators for feedback and refinement . Email newsletters are another means to distribute infographics to a targeted list of subscribers.A study was done on the characteristics required for good infographics. Based on a multi-method approach, which included eye tracking, memorability, and user perception, the authors categorised the infographic as good, average, and poor. The infographics are also helpful in research dissemination and medical education on social media.

The tools for creating infographics commonly employed are PowerPoint and Google Slides. These tools outline the content by defining the aim, the main message (should contain only critical information), and the type of audience. The abstract should contain the process, progress, and statistics, and the content should encourage the reader to act. The conclusion should carry a take-home message. The steps in creating graphics are content, layout, platform, colour, and feedback [55].

### 1.2. Significance of the Study

This study is significant because it addresses the gap between the rapidly growing volume of medical data and the need for effective communication, which continues to generate an exponential volume of data and insights. Infographics offer a dynamic solution capable of rendering intricate medical concepts understandable to a broader audience, including policymakers, patients, and the public. The public is often nonspecialist, audiences that stand to benefit from its findings.

This study highlights the importance of using infographics and graphical abstracts for user engagement, retention, and understanding. The findings help us understand the existing use of infographics by health professionals. The insights gained here could catalyse a shift in how medical researchers approach knowledge dissemination, encouraging the integration of innovative visualisation techniques to amplify the impact of their findings. Furthermore, this research presents an opportunity to shed light on the most effective platforms and channels for disseminating infographics in the medical domain. By uncovering the preferred modes of information delivery among medical professionals, this study has the potential to inform strategic decisions for research communication, facilitating a more effective reach to target audiences.

## 2. Methods

The objectives of this study are as follows:

- Assessing familiarity with infographic design and data visualisation principles
- Evaluating the effectiveness of infographics for research dissemination
- Examining tools and practices for creating infographics in research dissemination
- Identifying the data types most suitable for infographic presentation
- Determining the primary platforms or channels for sharing infographics in research dissemination

### 2.1. Research Design

This study employed a cross-sectional survey design to gather data from medical professionals who had published research articles. Most of the participating researchers were from Asian countries, and they distributed the questionnaire via Web 2.0 to colleagues in various other countries. The participants were authors who had published articles in PubMed and Scopus without restrictions on the number of articles or specific journals. This study included authors from any medical field who held a master's or doctorate and were working in universities or public/private sectors, provided they published at least one article per year in Scopus or PubMed.

The self-evaluated questionnaire, designed by the researchers, consisted of Likert-scale questions and demographic variables to capture respondents' characteristics and opinions regarding infographics.

### 2.2. Questionnaire Preparation and Validation

The questionnaire was developed by reviewing existing literature on infographics, data visualisation, and research dissemination. Initial items were formulated to measure respondents' familiarity, perceptions, and practices related to infographics. The questionnaire was reviewed by experts in medical communication, research methodology, and survey design to ensure content validity. Feedback from experts was used to refine and revise the questionnaire.

A pilot study was conducted with a small sample of medical professionals to assess the questionnaire items' clarity, relevance, and comprehensibility. Feedback from the pilot study participants was collected through open-ended responses and used to further refine the questionnaire.

### 2.3. Participants

The survey respondents included medical professionals with a history of published research articles. The study targeted professionals from various medical subfields, including but not limited to Clinical Medicine, Epidemiology, Pharmacology, Healthcare Management, Medical Imaging, and Public Health.

### 2.4. Data Collection

E-mail addresses were obtained from their published articles. Data were collected using an online survey distributed to potential participants through professional networks and research institutions. The survey comprised a refined questionnaire that explored respondents' familiarity with infographic design, perceptions of infographic effectiveness, favoured tools for crafting infographics, data types deemed suitable for infographic presentation, and preferred platforms for disseminating infographics.

The survey commenced with providing voluntary consent to participants, outlining the study's objectives, their rights, and the intended use of data. Demographic details such as gender, age, professional experience, and geographic location were gathered to establish a comprehensive profile of the respondents.

### 2.5. Data Analysis

Descriptive statistics analysed respondents' demographic characteristics and familiarity with infographic design and data visualisation principles. Mean, standard deviation, t values, and *p* values were computed to assess respondents' perceptions of different aspects of infographic design. The percentages of responses were calculated for aims, metrics, factors, tools, and channels related to infographics.

### 2.6. Ethical Considerations

Ethical approval was obtained from the relevant institutional review board to ensure participant consent and data protection. Participation in the survey was voluntary, and respondents' anonymity and confidentiality were maintained throughout the study. The collected data were securely stored and used solely for research purposes.

## 3. Results

Table 1 shows the demographic details of the study respondents. The survey included 339 medical professionals, with a majority being male (58.7%). Most respondents were aged between 35 and 54 years (67.3%), indicating midcareer professionals. The participants had substantial experience, with 66.9% having over 10 years in their field. Geographically, the survey was diverse, with a significant representation from Asia (43.4%), followed by Europe (18%) and Africa (14.4%). North and South America contributed 7.4% and 12.7% of respondents, respectively, while Oceania had 4.1%. These data reflect a well-experienced, internationally diverse group, predominantly from Asia, and highlight a gender imbalance favouring male respondents.

Table 2 assesses respondents' familiarity with infographic design and data visualisation principles. Participants rated various aspects on a scale from 1 (low familiarity) to 5 (high familiarity). The mean scores and standard deviations (SDs) indicate respondents' perceptions. Notably, respondents found conveying messages and storytelling through data visualisation (mean = 3.98) and techniques for visualising numerical data (mean = 3.72) areas of higher familiarity. Conversely, highlighting patterns, trends, and relationships in data (mean = 2.94) received a lower familiarity rating. Statistical analysis showed no significant differences (*p* > 0.05) in respondents' familiarity with the assessed principles.

Table 3 illustrates the perceived effectiveness of infographics in research dissemination, outlining the aims and outcomes identified by respondents. Among 339 participants, the majority believed that using infographics could achieve the following aims: increasing engagement with research findings (94.1%), simplifying complex information (95.9%), enhancing visual appeal (87.9%), increasing social media sharing (80.8%), improving accessibility and reaching a broader audience (69.3%), highlighting critical insights and trends (90%), presenting data memorably and compellingly (76.1%), facilitating data-driven decision making (73.4%), promoting information retention (74.9%), and providing a quick overview of research findings (93.8%). A small portion (1.2%) marked N/A for this assessment. These responses collectively demonstrate the multifaceted benefits of infographics in research dissemination.

Table 4 outlines the methods and metrics used to evaluate the efficacy of infographics in research dissemination based on responses from 339 participants. The assessment approaches include user feedback (71.1%), social media engagement metrics (63.1%), website analytics (53.4%), survey responses from the target audience (54%), time spent interacting with the infographic (48.7%), number of citations or references in academic works (58.7%), e-mail or direct message responses (29.8%), conversion rates for desired actions (84.7%), comparison against other dissemination methods (26.3%), and expert review of clarity and effectiveness (75.2%). These diverse evaluation techniques demonstrate a comprehensive approach to gauging the impact and value of infographics in research communication. The graphics assisted the researchers in better visualisation and reading from other researchers and for the public.

Table 5 outlines the key elements for successful research infographic dissemination based on responses from 339 participants. These factors are vital for effective communication. Participants identified the following factors as crucial: clear and concise content (97.3%), engaging visuals and graphics (93.2%), effective data visualisation techniques (81.1%), appropriate colour schemes and fonts (79.6%), incorporation of relevant charts, graphs, or diagrams (87%), attention to design principles and aesthetics (44%), alignment with the target audience's preferences and needs (46.9%), proper use of hierarchy and visual organisation to guide attention (57.5%), consistent and cohesive visual style (42.5%), and incorporation of storytelling elements for relatability and memorability (93.8%). These findings underscore the significance of a balanced integration of design and content factors to ensure impactful research infographic dissemination. The poor alignment of the observation with audience needs could have been due to being unable to extract key findings, assuming that the audience is already aware, misinterpretation, inadequate contextualization, lack of interaction, unappealing design, or ignoring audience preferences. Feedback from the intended audience during the pilot study can avoid these problems.

Table 6 presents the software and tools employed for creating infographics in research dissemination, as reported by 339 participants. The breakdown includes Adobe Illustrator (51.3%), Canva (57.5%), Piktochart (46.9%), PowerPoint (72.3%), Excel (44%), Adobe Photoshop (39.5%), Adobe InDesign (24.8%), Microsoft Word (64.9%), Venngage (25.1%), Visme (41%), Infogram (23.6%), Snappa (19.2%), and Google Slides (70.5%). A small portion indicated “N/A” (7.1%) for this question. This diverse array of tools showcases the variety of options researchers use to create impactful and engaging infographics for research dissemination.

Table 7 provides insights into the types of data considered suitable for presentation in infographics, as indicated by 339 participants. The data types and corresponding percentages are as follows: statistical data (73.5%), comparative data (55.8%), time series data (42.2%), geographic data (69.9%), categorical data (31.9%), medical imaging data (78.2%), healthcare costs and expenditure (50.1%), clinical trial results (87%), epidemiological data (43.7%), drug and treatment comparisons (58.4%), and a minority marking “N/A” (12.7%). This diverse range of data types demonstrates the versatility of infographics in effectively conveying various types of research information.

Table 8 highlights the primary platforms or channels selected for sharing infographics in research dissemination based on responses from 339 participants. The distribution is as follows: social media platforms (51.6%), research blogs or websites (42.2%), online research repositories (33.9%), e-mail newsletters or mailing lists (28%), conference presentations or posters (50.1%), science communication platforms (87.9%), institutional or departmental websites (58.7%), slide-sharing platforms (59.3%), research collaboration platforms (28%), video-sharing platforms for animated or interactive infographics (33%), and a minority choosing “N/A” (11.2%). This assortment of dissemination channels underscores researchers' varied approaches to effectively reaching their intended audiences with research infographics.

## 4. Discussion

In our study, the data indicated familiarity with infographics among medical professionals, and all agreed that infographics are the most effective in comprehension. However, when it came to using infographics practically, most needed more familiarity. On a scale of 1 to 5, the maximum familiarity of 3.98 agreed that messages with data visualisation were read more. The lowest score of 2.94 was that they needed more familiarity with highlighting patterns, trends, and relationships in data through visualisation. The majority also needed to gain more familiarity, 3.04, in knowledge of best practices in designing infographics and the types of infographics used. The other areas of low familiarity included accurately representing the underlying data in data visualisations and ensuring accessibility to broad audiences. Hence, the above data confirm that trained designers should be included in the team to create infographics. The infographics can be perfected by an expert review team, including medical professionals and patients, to evaluate if the information conveyed is the same as the intended information [57].

Regarding the effectiveness of infographics in research dissemination, 95.9% agreed that the main aim of using graphics is to simplify complex information, followed by enhanced research findings. Respondents consider the infographics to be a quick overview of data highlighting critical insights and trends (90%). Only 74–76% agreed that infographics present data compellingly and with good retainability for data-driven decision making. All agreed that data accessibility needs to be improved for a broader audience. The most common form of infographic research dissemination is for evaluations, to take desired actions, feedback, social media engagement, website analytics, and survey responses from the audience. The lowest response, 26.3%, was for comparing infographic performance against other research dissemination methods. Besides the above uses, infographics are equally valuable in teaching students [58]. Various medical journals have graphical abstracts that enable the reader to scan recent research articles, provide more readability, and provide significant points for critical decision making [59].

Among the tools used to create infographics, PowerPoint was the most used tool, 72.3%, followed by Google Slides, Microsoft Word, Canva, Adobe Illustrator, Piktochart, Excel, and Adobe Photoshop. Adobe InDesign, Venngage, Visme, Infogram, and Snappa were the less frequently used tools. Digital tools used by students are also termed mind tools. Digital tools such as Piktochart need semiautomation as authors can select from various templates available. Mastering digital tools is easy, as most digital tools assist with tutorial videos [60].

Among medical professionals, the most familiar data shared by infographics are clinical trial results from 87%, followed by medical imaging data from 78.2%. The other data are statistical data at 73.5%, geographical data at 69.9%, and drug and treatment comparison at 58.4%. The rest of the data shared below 50%, as per our findings, were healthcare cost and expenditure, epidemiological data, and time series data. Infographics have been successfully used in libraries, educational institutions, marketing, business, medicine, and media [2, 61, 62].

The platforms used commonly to share medical data, ResearchGate and Academia.edu, were observed to be 87.9%. The other platforms to share research data were conferences, poster presentations, websites, research blogs, slide-sharing platforms, and research collaboration platforms. Additionally, reference management tools such as Mendeley and Zotero played a significant role in organizing and sharing research findings. Online repositories such as Figshare, Zenodo, Prospero, and Cochrane were reliable platforms. Video-sharing platforms such as YouTube, Vimeo, Instagram, Twitter, Pinterest, and Facebook are also platforms that the majority use to share knowledge in visual form.

### 4.1. Limitations of the Study

The data collected from our observations were the result of participants' self-reporting and are not supplemented with any measure of effectiveness or data about how the recipients of the research knowledge/infographics viewed the material, the style of presentation, and how it impacted their knowledge or practice. The only method of evaluation was the answers to the questionnaire. This study implies a linear dissemination process and requires a more dynamic knowledge translation and dissemination. Infographics are likely one tool in various methods for translating knowledge into practice and policy.

## 5. Conclusion

This cross-sectional study among medical professionals revealed familiarity with infographic design and data visualisation principles. They recognise the effectiveness of infographics in achieving multiple aims, including increasing engagement, simplifying complex information, enhancing visual appeal, and improving accessibility. Respondents also identified clear and concise content, engaging visuals, and effective data visualisation techniques essential for successful research infographic dissemination. In terms of tools and practices, the research unveiled a diverse array of software and tools used by medical professionals for creating infographics, including popular options such as PowerPoint, Adobe Illustrator, Canva, and Microsoft Word. This diversity highlights the flexibility and adaptability of researchers in choosing the tools that best suit their needs.

Regarding data types suitable for infographic presentation, the study found that medical professionals consider a wide range of data types, including statistical data, medical imaging data, clinical trial results, and more, suitable for presentation in infographics. This suggests that infographics can effectively convey several types of medical research information. In the realm of dissemination, the study identified many platforms and channels medical professionals use to share infographics. Social media, science communication platforms, and institutional websites emerged as prominent choices, reflecting the importance of online and digital channels in modern research communication.

Future research should include the evaluation of evidence-based literature and how many infographics were used to convey information. The wider practice of using infographics, familiarity with infographics, preferences for data types, and dissemination practices will be evaluated. Additionally, longitudinal studies could assess the evolving trends and adoption rates of infographics in medical research dissemination over time. Moreover, investigating the impact of infographics on patient education, healthcare decision making, and research translation into practice could provide valuable insights into the real-world implications of using infographics in the medical field.

## Figures and Tables

**Table 1:** Demographic and professional characteristics of survey respondents.

Demographics	Statement	Respondents	Percentage
Gender	Male	199	58.7
	Female	140	41.3

Age	25–34	21	6.2
	35–44	123	36.3
	45–54	105	31
	55–64	62	18.3
	65 or older	28	8.2

Professional experience	Less than 1 year	2	0.6
	1–5 years	42	12.4
	6–10 years	90	26.5
	11–15 years	135	39.8
	More than 15 years	70	20.6

Geographic location	North America (Canada (8), Mexico (4), United States (10), and Jamaica (3))	25	7.4
	South America (Argentina (14), Brazil (15), Bolivia (7), Chile (2), and Colombia (5))	43	12.7
	Europe (Germany (4), Sweden (8), Denmark (10), Norway (11), United Kingdom (8), France (4), Poland (2), Austria (5), Croatia (4), and Ireland (5))	61	18
	Asia (India (51), Indonesia (11), Thailand (33), Saudi Arabia (11), Malaysia (5), Sri Lanka (3), and United Arab Emirates (33))	147	43.4
	Africa (Nigeria (7), Ethiopia (5), Tanzania (9), South Africa (4), Kenya (6), Malawi (5), and Zambia (13))	49	14.4
	Oceania (Australia (8), New Zealand (4), and Fiji (2))	14	4.1

**Table 2:** Assessing familiarity with infographic design and data visualisation principles.

Familiarity with infographic design and data visualisation techniques	Mean	SD	*T* value	*p* value
Visually appealing and engaging infographics	3.28	1.04	0.004	0.997
Organising and structuring information in infographics	3.59	1.04	0.149	0.883
Avoiding common mistakes in infographic creation	3.7	1.07	0.138	0.891
Purpose and design differences of infographics in research presentations	3.26	1.09	0.066	0.949
Commonly used infographics in research presentations	3.26	1.09	0.13	0.897
Best practices for designing infographics in your discipline	3.04	1.12	0.019	0.985
Choosing appropriate colours and fonts in infographics	3.55	1.03	0.08	0.938
Ensuring accessibility of infographics to a wide range of audiences	3.35	0.95	0.021	0.984
Techniques for visualising numerical data in the infographic	3.72	0.93	0.18	0.858
Incorporating charts, graphs, and visual elements into infographics	3.49	1.01	0.152	0.88
Accurately representing underlying data in data visualisations	3.19	1.02	0.133	0.895
Avoiding common pitfalls in data visualisation	3.44	1.11	0.037	0.971
Conveying messages and storytelling through data visualisation	3.98	0.96	0.046	0.964
Selecting appropriate data visualisation techniques for different data types	3.27	1.09	0.067	0.948
Highlighting patterns, trends, and relationships in data through visualisation	2.94	1.1	0.06	0.953

**Table 3:** Effectiveness of infographics in research dissemination: aims and outcomes.

When using infographics for research dissemination, the aims can include	Respondents	Percentage (*N* = 339)
Increase engagement with research findings	319	94.1
Simplify complex information	325	95.9
Enhance visual appeal	298	87.9
Increase social media sharing	274	80.8
Improve accessibility and reach a broader audience	235	69.3
Highlight critical insights and trends	305	90
Present data memorably and compellingly	258	76.1
Facilitate data-driven decision making	249	73.4
Promote information retention	254	74.9
Provide a quick overview of the research findings	318	93.8
N/A	4	1.2

**Table 4:** Evaluating the efficacy of infographics in research dissemination: metrics and assessment methods.

Assessing infographic effectiveness	Respondents	Percentage (*N* = 339)
User feedback	241	71.1
Social media engagement (likes, shares, comments)	214	63.1
Website analytics (page views, click-through rates)	181	53.4
Survey responses from the target audience	183	54
Time spent interacting with the infographic	165	48.7
Number of citations or references to the infographic in academic papers or articles	199	58.7
E-mail or direct message responses from viewers	101	29.8
Conversion rates of viewers to take desired actions (e.g., signing up for a newsletter, downloading a report)	287	84.7
Comparison of infographic performance against other research dissemination methods	89	26.3
Expert review and evaluation of the infographic's clarity and effectiveness	255	75.2

**Table 5:** Key elements for successful dissemination of research infographics: design and content factors.

Factors for effective research infographic dissemination	Respondents	Percentage (*N* = 339)
Clear and concise content	330	97.3
Engaging visuals and graphics	316	93.2
Effective data visualisation techniques	275	81.1
Use of appropriate colour schemes and fonts	270	79.6
Incorporation of relevant charts, graphs, or diagrams	295	87
Attention to design principles and aesthetics	149	44
Alignment with the target audience's preferences and needs	159	46.9
Proper use of hierarchy and visual organisation to guide the viewer's attention	195	57.5
The consistent and cohesive visual style throughout the infographic	144	42.5
Incorporation of storytelling elements to make the information more relatable and memorable	318	93.8

**Table 6:** Examining tools and practices for creating infographics in research dissemination.

Software/tools for infographic creation	Respondents	Percentage (*N* = 339)
Adobe Illustrator	174	51.3
Canva	195	57.5
Piktochart	159	46.9
PowerPoint	245	72.3
Excel	149	44
Adobe Photoshop	134	39.5
Adobe InDesign	84	24.8
Microsoft Word	220	64.9
Venngage	85	25.1
Visme	139	41
Infogram	80	23.6
Snappa	65	19.2
Google Slides	239	70.5
N/A	24	7.1

**Table 7:** Identifying the data types suitable for infographic presentation.

Types of data suitable for presentation in infographics	Respondents	Percentage (*N* = 339)
Statistical data	249	73.5
Comparative data	189	55.8
Time series data	143	42.2
Geographic data	237	69.9
Categorical data	108	31.9
Medical imaging data	265	78.2
Healthcare costs and expenditure	170	50.1
Clinical trial results	295	87
Epidemiological data	148	43.7
Drug and treatment comparisons	198	58.4
N/A	43	12.7

**Table 8:** Determining the primary platforms or channels for sharing infographics in research dissemination.

Platforms/channels for sharing infographics in research dissemination	Respondents	Percentage (*N* = 339)
Social media (e.g., Facebook, Twitter, LinkedIn)	175	51.6
Research blogs or websites	143	42.2
Online research repositories (e.g., Figshare, Zenodo)	115	33.9
E-mail newsletters or mailing lists	95	28
Conference presentations or posters	170	50.1
Science communication platforms (e.g., ResearchGate, Academia.edu)	298	87.9
Institutional or departmental websites	199	58.7
Slide-sharing platforms (e.g., SlideShare, Google Slides)	201	59.3
Research collaboration platforms (e.g., Mendeley, Zotero)	95	28
Video-sharing platforms (e.g., YouTube, Vimeo) for animated or interactive infographics	112	33
N/A	38	11.2

## Data Availability

Data are available on reasonable request by contacting subaveerapandiyan@gmail.com.
